# Supplementary material for: Antimicrobial Activities of α-Helix and β-Sheet Peptides against the Major Bovine Respiratory Disease Agent, Mannheimia haemolytica
Source: Int J Mol Sci. 2024 Apr 9;25(8):4164. doi: 10.3390/ijms25084164 (PMC11050306; doi:10.3390/ijms25084164)
Supplement: Supplementary file 1 [file ijms-25-04164-s001.zip › Supplemental Figure S1.pdf]

Peptide MIC Plate map

		1	2	3	4	5	6	7	8	9	10	11	12
Peptide (µg/mL)		256	128	64	32	16	8	4	2	1	0.5	0.25	0
Mh 535	A												
Mh 535	B												
Mh 587	C												
Mh 587	D												
Mh 13	E												
Mh 13	F												
E. coli ATCC25922	G												
Media diluent	H												

Figure S1: Plate layout for determining the minimum inhibitory concentration of antimicrobial peptides against *M. haemolytica.*
