# Supplementary material for: Antimicrobial Activities of α-Helix and β-Sheet Peptides against the Major Bovine Respiratory Disease Agent, Mannheimia haemolytica
Source: Int J Mol Sci. 2024 Apr 9;25(8):4164. doi: 10.3390/ijms25084164 (PMC11050306; doi:10.3390/ijms25084164)
Supplement: Supplementary file 1 [file ijms-25-04164-s001.zip › Supplemental Figure S2.pdf]

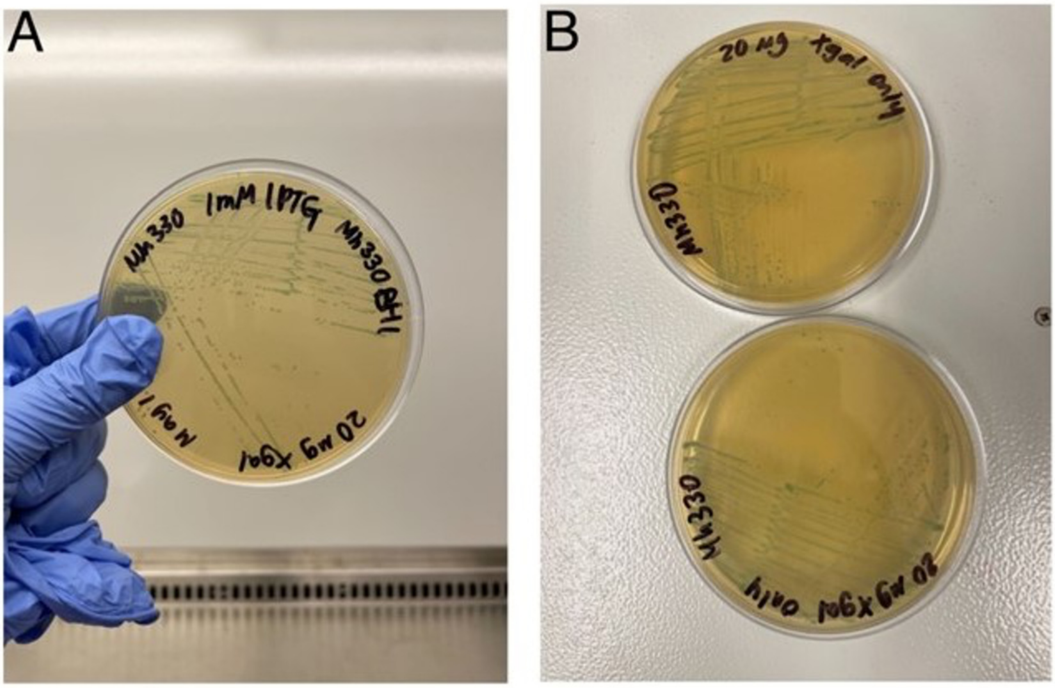

Figure S2. (A) *M. haemolytica* Mh 330 streaked on BHI agar with 1 mM IPTG and 20 µg of X-gal (B) Mh 330 streaked on BHI agar with X-gal only
